# NEAT1 variant 1 weakens the genome-wide effect of miR-3122 on blocking H3K79me3 in bladder cancer

**DOI:** 10.18632/aging.204113

**Published:** 2022-06-10

**Authors:** Wenchao Zhao, Fanghao Sun, Liansheng Zhang, Jun Ouyang

**Affiliations:** 1 Department of Urology, The First Affiliated Hospital of Soochow University, Suzhou, Jiangsu, China

**Keywords:** NEAT1, variant, miR-3122, H3K79me3, bladder cancer

## Abstract

Nuclear-enriched abundant transcript 1 (NEAT1) is one of the most well-studied long non-coding RNAs (lncRNAs) in multiple human carcinoma. Two distinct variants of NEAT1, however, are never illuminated their specific functions and mechanisms underlying carcinogenesis. In this study, biotin-labelled NEAT1 variants were generated to incubate with cell lysate of bladder cancer cell T24 cells, and fished a batch of RNA substances. Here, we observed that NEAT1.1 (the short transcript) could capture 122 microRNAs (miRNAs), 36 small nucleolar RNAs (snoRNAs), 55 lncRNAs and 38 mRNAs while NEAT1.2 (the long transcript) could obtain 142 miRNAs, 51 snoRNAs, 72 lncRNAs and 41 mRNAs. Furthermore, we also found that the distinctions of RNA binding substances between these two variants were mainly expressed in nucleus rather than cytoplasm. GO analysis indicated that these non-coding RNAs governed histone modification, nucleosome assembly and chromosome organization. We picked up miRNA miR-3122, which substantially interacted with NEAT1.1, and found that histone H3K79me3 was reduced in bladder cancer T24, BIU-87 and EJ-1 cells after miR-3122 overexpression, and rescued by NEAT1.1 additional compensation. Nonetheless, we failed to find that miR-3122 could interfere with expression of H3K79 methyltransferase disruptor of telomeric silencing-1 like (DOT1L). Interestingly, we harvested histone 3 fished by biotin-labelled miR-3122, and validated this intercrossing using RNA immunoprecipitation. Taken together, we demonstrated that NEAT1.1 weakened the effect of miR-3122 on H3K79me3 suppression in bladder cancer.

## INTRODUCTION

Bladder cancer (BC), one of the most common urogenital tumors, presents high incidence, prevalence, recurrence and mortality [1, 2]. The multiple factors are involved in etiology of BC, including genetic, epigenetic and environmental factors. The conventional therapies of BC start with surgical resection followed by adjuvant therapies, such as radiotherapy and chemotherapy. The adjuvant therapies are introduced in particular when the tumor appears loco-regional and distant invasion. Effective chemotherapeutic and targeted drugs were introduced to systematically advance the treatment options in BC over the last two decades. However, the tumor resistance to therapy is still a major challenge in individual treatment with low toxicity and significant benefit. Tumor can become tolerant to these pharmaceutical treatments including resistance emerges as well as early tumor recurrence.

Emerging evidence shows that long non-coding RNAs (lncRNAs) have attracted more and more attention as one of the epigenetic regulatory factors and potential therapeutic targets for multiple cancers [3]. One lncRNA named nuclear paraspeckle assembly transcript 1 (NEAT1) has been widely acknowledged to play a crucial role in malignancy on BC and other diseases [4–6]. NEAT1 participates in multiple biological pathways through a diverse group of mechanisms due to the formation of the paraspeckle, which it can influence the stability of a tumor to develop chemoresistance [7]. Interestingly, two existing variants of NEAT1 serve the opposite effects on the capabilities of tumor growth, apoptosis and invasion. Knockdown of NEAT1.1 (the short transcript) *in vitro* could inhibit cell invasion and proliferation [8], while knockdown of NEAT1.2 (the long transcript) promoted cell growth [9]. One explanation is the architectural functions in the construction of the nuclear body [10]. However, the complicated regulatory network of different NEAT1 variants for BC tumorigenesis is not fully understood.

In this study, we produced full length of NEAT1.1 and 1.2 with biotin tag, and fished the interacted RNAs. We analyzed them and finally focused on the epigenetic function of miR-3122 in BC cells. Our research reported a novel regulatory mechanism of NEAT1 in BC tumorigenesis, and provided an example of epigenetic study on other lncRNAs expressed in nucleus.

## RESULTS

### Binding RNA profiling of NEAT1 in BC cells

**Figure 1:**
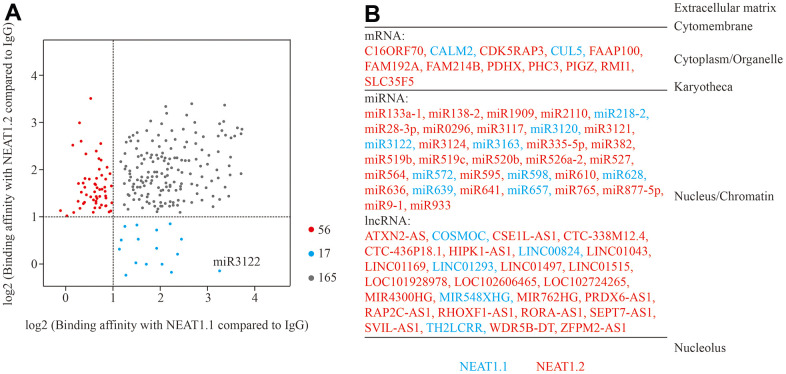
**Binding RNA profiles of NEAT1 in T24 BC cells.** (**A**) Grey spots represent RNAs which significantly interact with NEAT1.1 and NEAT1.2 compared to blank biotin IgG (log_2_FC > 1, *p* < 0.05). Blue spots represent the ones only interact with NEAT1.1, whereas red spots are only for NEAT1.2. The spot of miR-3122 is highlighted. (**B**) The different categories of binding RNAs are listed according to the cellular localization. The spots in (**A**) are listed in red and blue fonts accordingly.

First, we incubated biotin-labelled NEAT1.1 and NEAT1.2 with cell lysate of bladder cancer cell T24 cells, and performed RNA pull down and next generation sequencing to investigate the binding RNAs with NEAT1. Compared to the negative control of unmatched RNA with biotin, data showed a series of binding RNA with NEAT1. We observed that NEAT1.1 could capture 85 miRNAs, 55 lncRNAs, 39 mRNAs and 3 other RNAs, while NEAT1.2 could obtain 101 miRNAs, 72 lncRNAs, 45 mRNAs and 3 other RNAs (log_2_FC > 1, *p* < 0.05). Although 3.7 kb NEAT1.1 was completely included in 22.7 kb NEAT1.2, the binding RNAs were still different. In total 73 RNAs showed the binding distinction between NEAT1.1 and NEAT1.2 (Figure 1A). In this list of differential binding RNAs, nearly all of them were expressed in nucleus and chromatin (Figure 1B) according to database of “RNALOCATE” [11]. Furthermore, we also found that the distinctions of RNA binding substances between these two variants were mainly expressed in nucleus rather than cytoplasm. In summary, we characterized the binding RNA profiles of NEAT1 in BC T24 cells.

### The intercrossing between miR-3122 and NEAT1.1

**Figure 2:**
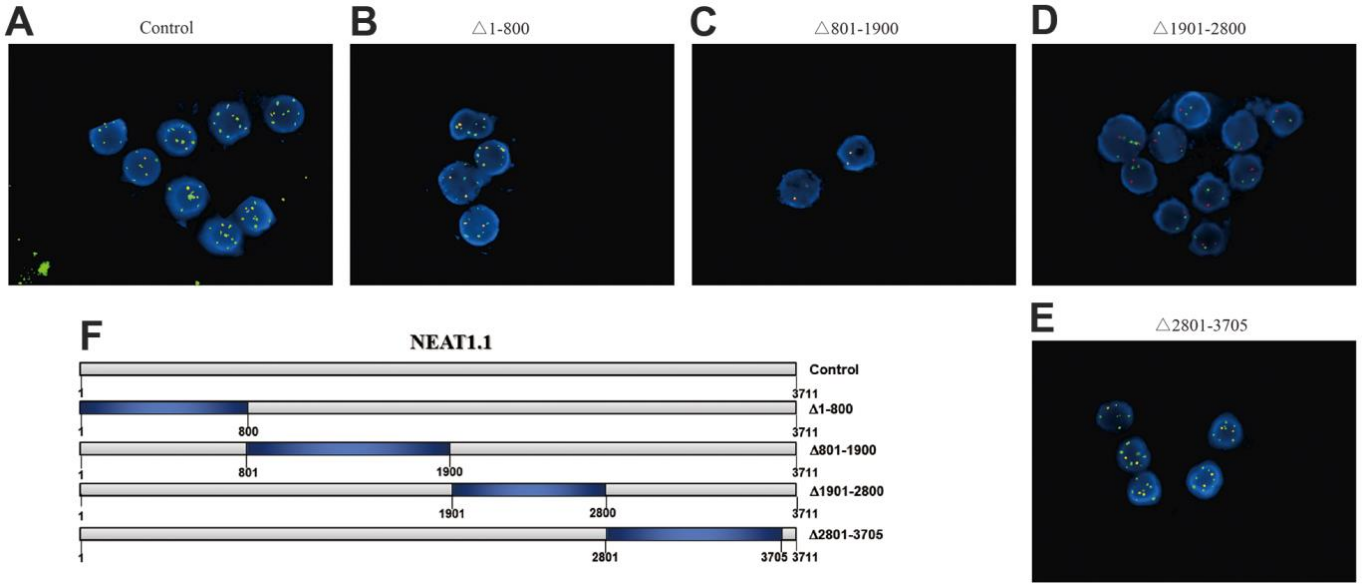
**The interaction between NEAT1.1 and miR-3122.** FISH assay shows the contacts with miR-3122 and wild type NEAT1.1 (**A**), NEAT1.1 with 1-800 truncation (**B**), 801-1900 truncation (**C**), 1901-2800 truncation (**D**), 2801-3705 truncation (**E**) in T24 cells with 200 x magnification. Red signals indicate NEAT1.1, and green signals indicate miR-3122. Yellow signals mean the overlapping of these two probes. (**F**) The schematic representation of NEAT1.1 with truncation of each fragment.

Next, we picked up miR-3122, which had the most significant in differential binding RNAs (*p* = 3.4x10^-8^). Our FISH assay validated that miR-3122 and NEAT1.1 had a substantial interaction with each other instead of NEAT1.2 (Figure 2A). Furthermore, we also determined that miR-3122 could bind with 1901 to 2800 of NEAT1.1 (Figure 2B–2E) as we prepared the different truncation of NEAT1.1 (Figure 2F). Taken together, we demonstrated the intercrossing between miR-3122 and NEAT1.1.

### Mono-methylation of H3K79 inhibited by miR-3122

**Figure 3:**
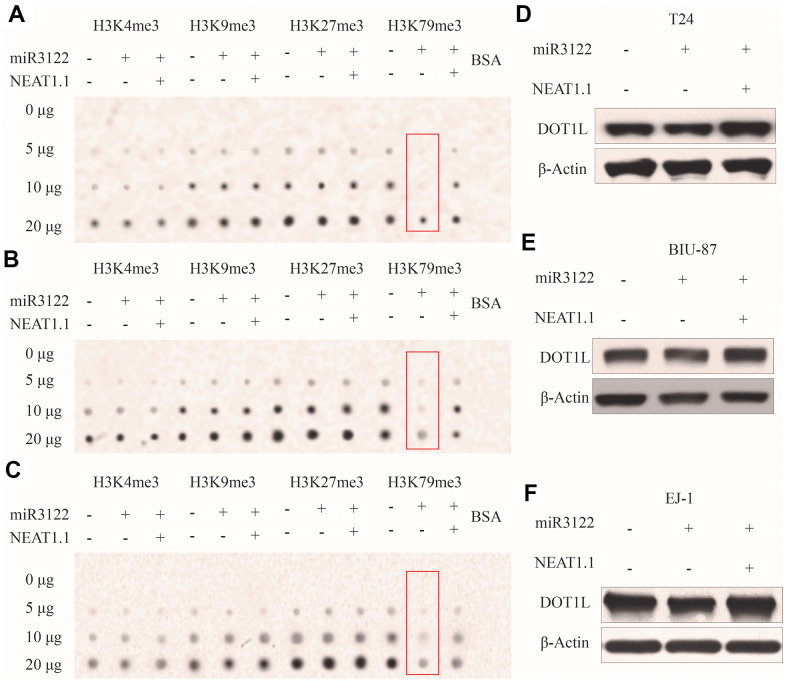
**miR-3122 affects global H3K79me3 in BC cells.** Dot plot assay show the global levels of H3K4me3, H3K9me3, H3K27me3 and H3K79me3 in T24 (**A**), BIU-87 (**B**) and EJ-1 (**C**) cells with ectopic miR-3122 and NEAT1.1 over-expression. The expression of H3K79 methyltransferase DOT1L in T24 (**D**), BIU-87 (**E**) and EJ-1 (**F**) cells with ectopic miR-3122 and NEAT1.1 over-expression.

We had found that miR-3122 was mainly expressed in nucleus, it did not look like regular studies looking for mRNA targets in the cytoplasm. To investigate the epigenetic role of miR-3122 in nucleus of BC cells, we performed dot plot assay to study the histone modifications. From the pattern of histone H3 methylation, we unexpectedly observed that histone H3K79me3 was significantly reduced after miR-3122 overexpression, and rescued by NEAT1.1 additional compensation in T24 cells (Figure 3A), which was further verified in BIU-87 and EJ-1 cells (Figure 3B, 3C). Nonetheless, we failed to find that miR-3122 could interfere with expression of H3K79 methyltransferase disruptor of telomeric silencing-1 like (DOT1L) (Figure 3D–3F).

**Figure 4:**
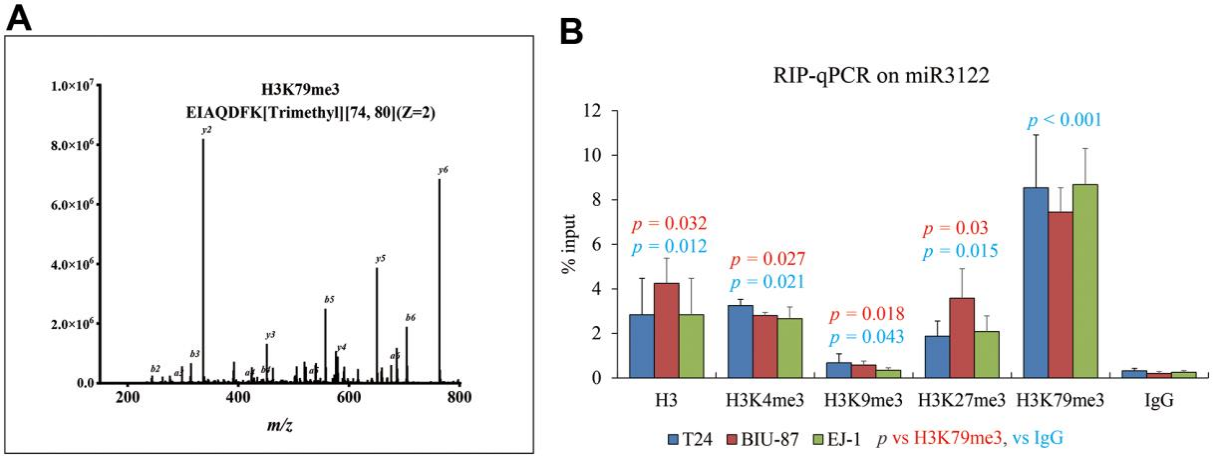
**miR-3122 enriches the histone H3 with robust H3K79me3 modification.** (**A**) Representative MS2 spectra shows H3 protein in T24 cells. Inset: Fragmentation patterns of b and y ions show sequence information, amino acid residue, m/z and charge state identified by LC-MS/MS. (**B**) RIP-qPCR assay shows the enrichment of miR-3122 in histone 3 with different lysine methylation in T24, BIU-87 and EJ-1 BC cells. The *p*-values represent the statistical significance compared with K3K79me3 or IgG according to different color fonts via One-way ANOVA analysis.

Similarly with molecular fishing of NEAT1, biotin-labelled miR-3122 was used to search the binding protein by mass spectrum in T24 cells, and we harvested histone H3 as one of the top plausible binding proteins with K79 trimethylation (Figure 4A). Consistently, RNA immunoprecipitation assay was conducted to validate the intercrossing between miR-3122 and histone H3 in T24, BIU-87 and EJ-1 cells. We found that H3K79me3 could obtain the highest enrichment of miR-3122 compared to other H3 histone modifications (Figure 4B), indicating that H3K79me3 was attributed to miR-3122. Overall, we determined that miR-3122 would affect genome-wide H3K79me3 to regulate gene transcription.

## DISCUSSION

NEAT1 is a well-acknowledged lncRNA functioning in development and diseases via affecting multiple signaling pathways. In the current massive literature, NEAT1 is entangled with various miRNAs in cytoplasm, even exosome. However, both of two NEAT1 variants are actually expressed in nucleus. NEAT1.2 contributes to building phase-separated nuclear paraspeckles via its high-affinity binding sites for recruitment of the essential paraspeckle proteins (NONO and SFPQ) [10, 12]. However, NEAT1.1 containing with only two domains for RNA stability and isoform selection seems to have contributed less to paraspeckle formation. The mechanism behind the opposite effects on tumor of NEAT1.1 and NEAT1.2 still remain obscure [13]. According to previous studies of the same kind non-coding RNAs in nucleus [14, 15], lncRNA could facilitate the connection between transcription factors or epigenetic regulators and DNA elements. In our research, our data unexpectedly supports that a large number of nucleus-expressed RNAs are enriched on NEAT1. Unlike the cytoplasm-expressed miRNA such as miR129-5p [16], miR214 [17], miR342-3p [18], miR141-3p [19], most of them especially in nucleus are less studied. We have no clue about the binding RNAs in other cancer, but the role of NEAT1 in BC needs to be re-examined.

The different binding RNAs between NEAT1 variants suggest that they NEAT1.1 has a distinct spatial structure from NEAT1.2 although the sequence of NEAT1.1 overlaps with the part of NEAT1.2. Taking miR-3122 in this study as an example, it can be shown that the miR-3122 binding domain of NEAT1.1 may be exposed to surface compared to NEAT1.2, indicating that lncRNAs served as sponging to miRNAs may be attributed to their alternative 2D even 3D structures.

So far, miRNA binding proteins are majorly centered around the argonaute (AGO)-family proteins-mediated miRNA-induced silencing complex [20, 21]. Emerging evidence indicating that other (RBPs) such as AUF1 and HuR can also directly interact with mature miRNAs [22, 23] also implicates the competitive binding of RBPs to target miRNAs, sequestration of miRNAs from AGO, promotion of AGO binding to miRNAs, and transfer of miRNAs from RBPs to AGO [24]. Although our data determines the coincidence of miR-3122 enrichment and H3K79me3 variation, we speculate that miR-3122 is supposed to bind with genomic DNA rather than histone 3 directly, and the binding regions packaged with nucleosome and controlling the transcription activity of target genes might have some unknown factors to recruit miR-3122. More experiments are needed to confirm this hypothesis in future study.

We summarize the binding RNAs of NEAT1.1 in BC cells, and find that NEAT1.1 can weaken the miR-3122-mediated suppressive effect on genome-wide H3K79me3. Our observations provide a brand-new action mode of lncRNA-miRNA for epigenetic regulation in cancer.

## MATERIALS AND METHODS

### Cell culture

Bladder cancer cell T24, BIU-87 and EJ-1 cells were purchased from Type Culture Collection of the Chinese Academy of Sciences (Beijing, China) were cultured in 1640 medium with 10% FBS (HyClone, USA) at the incubator with 37° C, 5% CO2 and 100% humidity.

### RNA synthesis *in vivo*

Full or truncated single-strand RNA products of NEAT1.1 and miR-3122 labelled by biotin were synthetized by Genepharma (Shanghai, China). Full length of NEAT1.2 was initially amplified by PCR using forward primer adding T7 promoter sequence. PCR products were purified as a template for RNA synthesis using T7 RNA polymerase kit (Cat. No. M0251L, NEB, USA). In the reaction system preparation, uridine nucleoside triphosphate (UTP) was replaced by the specific biotin labelled UTP (Cat. No. AM8451, Thermo Fisher Scientific, USA). DNase I (Cat. No. M0303S, NEB) was used for template removal, followed by ethanol precipitation. RNA products were examined the concentration and subpackaged to store at -80° C for the subsequent experiments.

### RNA pull down assay

400 μL streptavidin magnetic beads (Cat. No. S1420, NEB) for each experiment was washed by 1x binding & washing buffer (5 mM Tris-HCl, 0.5 M EDTA, 1 M NaCl, 0.025% Tween 20, 100 U/mL RNase inhibitor) for three times, then added 400 μL 2x binding & washing buffer and 2 μg RNA products for total 800 μL volume. The RNA-beads mixture was rotated gently for 20 min at room temperature, and washed again for three times, followed by cell lysis buffer (1 mM HEPES, 200 mM NaCl, 1% Triton X-100, 10 mM MgCl_2_, 1 mM DTT, 1x protease and phosphatase inhibitor cocktail, 1 U/μL RNase inhibitor) for resuspending to 500 μL [25, 26].

1×10^7^ cells were treated by 200 μL cell lysis buffer for 30 min on ice with the gently and repeatedly pipette-blowing, and added the freshly prepared RNA-beads mixture for 2-hrs gentle rotation at 4° C. After that, beads were washed by 400 μL cell lysis buffer for five times. For next generation sequence, beads were resuspended by 1 mL Trizol for RNA isolation. The final purified RNA was used for library construction. For mass spectrum, beads were resuspended by 1x protein loading buffer. Proteins were processed under metal bath at 100° C for 10 min, and the supernatant was transferred to a new tube without beads. Mass spectrum service is provided by Shanghai OEbiotech (Shanghai, China).

### Dot plot

1×10^7^ cells were treated by 400 μL cell lysis buffer containing 1x protease and phosphatase inhibitor cocktail for 30 min on ice with the gently and repeatedly pipette-blowing, then centrifuged with 14000 rpm for 15 min at 4° C. The total protein supernatant was determined the concentration, and dropped 2 μl each on nitrocellulose membrane with 2-fold serially dilution (0, 5, 10 and 20 μg) for dot blot assay. The membrane was dried and incubated with H3K4me3 (Cat. No. 61379, Activemotif, USA), H3K9me3 (Cat. No. 39285, Activemotif), H3K27me3 (Cat. No. 61017, Activemotif), H3K79me3 (Cat. No. NB21-1383, Novus, USA) for 4° C overnight with gentle shaking. The membranes were washed by TBST three times at room temperature, followed by rabbit anti-mouse or donkey anti-rabbit IgG-HRP (1:10000 dilution, Beyotime Biotechnology, China) in 10 ml of TBST for 1 h at room temperature with gentle shaking, and washed again for three times. After that membranes were incubated with 3 ml of ECL Western Blotting Substrate (Beyotime Biotechnology, China) for 5 min in darkness at room temperature and developed the spots.

### RNA immunoprecipitation assay

As previously described [27–32], 1×10^7^ cells were harvested, resuspended in nuclear isolation buffer (1.28 M sucrose, 40 mM Tris-HCl, 20 mM MgCl_2_, 4% Triton X-100) and kept on ice for at least 30 min with frequent mixing. The pellet nuclei were centrifuged with 14000 rpm for 15 min, resuspended by wash buffer (150 mM KCl, 25 mM Tris pH 7.4, 5 mM EDTA, 0.5 mM DTT, 0.5% NP40, 100 U/mL RNAase inhibitor, 1x protease and phosphatase inhibitor cocktail and sheared the chromatin through sonication by high power, 5 s on, 30 s off for 30 cycles. After that, 90% nuclei were incubated with 1 μg antibodies of H3K4me3, H3K9me3, H3K27me3 or H3K79me3 overnight and 40 μL Protein A/G beads (Thermo Fisher Scientific) 2 h by gentle rotation at 4° C while the rest of 10% were harvested as input. The pellet beads were centrifuged by 3000 rpm 3 min, washed three times. Both the input and pellet beads were purified by RNAiso plus (Takara, Japan) and conducted reverse transcription using QuantiTect Reverse Transcription Kit (Qiagen, Germany). Primer sequence for miR-3122: 5’- ACCAGCTCTGTTGGGACAAGAGGAC -3’; 5’- ACCAGCTCTCTTCGGACAAGATGA -3’.

### Western blot assay

1×10^7^ cells were treated by 400 μL cell lysis buffer containing 1x protease and phosphatase inhibitor cocktail for 30 min on ice with the gently and repeatedly pipette-blowing, then centrifuged with 14000 rpm for 15 min at 4° C. The total protein supernatant was determined the concentration. 40 μg total protein for each sample was added 1x protein loading buffer, and processed under metal bath at 100° C for 10 min, followed by sodium dodecyl sulfate–polyacrylamide gel electrophoresis (SDS–PAGE). Proteins were transferred to polyvinylidene fluoride membranes. The membranes were then blocked by 5% non-fat milk TBS for 90 min and then incubated with the respective primary antibodies of DTO1L (1:2000, Cat. No. NB100-40845, Novus) and β-Actin (1:5000, Cat. No. AF5001, Beyotime Biotechnology) overnight at 4° C. Membranes were washed in TBST and incubated with rabbit anti-mouse and goat anti-rabbit IgG-HRP at room temperature for 1 h. After that membranes were incubated with 3 ml of ECL Western Blotting Substrate for 5 min in darkness at room temperature and developed the bands.

### Fluorescence *in situ* hybridization assay

As previously described [33], the FISH probes for NEAT1.1 labelled with R-Phycoerythrin (excitation wavelength 565 nm), for miR-3122 labelled with FITC (excitation wavelength 495 nm) were purchased from G&P Medical Enterprises (Beijing, China). Slides filled with T24 cells washed by PBS were hypotension treatment in 0.075 M KCl at 37° C for 25 min, fixed by pure ethanol for 10 min and dropped on the glass slide, then aged at 56° C for 60 min. The slides were washed by PBS twice and dehydrated with the 70%, 85% and 100% ethanol for 3 min in order, and naturally dried. 10 μl probes were added on and mounted immediately. Slides were next denatured 75° C for 5 min and 42° C for 16 h, then removed the cover glass and incubated in 2 × SSC for 5 min and 0.1% NP-40/2 × SSC for another 2 min at 46° C, followed by 70% ethanol at room temperature for 3 min and dried in dark. 15 μl DAPI were dropped and mounted again. After 10 min incubation in dark, the slides were observed under the fluorescence microscope with the appropriate filters.

### Statistical analysis

All experimental data were processed and analyzed using SPSS 22.0 statistical software. The measurement data were expressed as mean ± standard deviation. One-way ANOVA was used for comparison between multiple groups. The *p*-value less than 0.05 was considered as statistical significance.

## References

[1] Yang C, Mou Z, Zhang Z, Wu S, Zhou Q, Chen Y, Gong J, Xu C, Ou Y, Chen X, Dai X, Jiang H. Circular RNA RBPMS inhibits bladder cancer progression via miR-330-3p/RAI2 regulation. Mol Ther Nucleic Acids. 2021; 23:872–86. 10.1016/j.omtn.2021.01.009 33614236 PMC7868720

[2] Chen L, Li W, Li Z, Song Y, Zhao J, Chen Z, Kazobinka G, Li L, Xing Y, Hou T. circNUDT21 promotes bladder cancer progression by modulating the miR-16-1-3p/MDM2/p53 axis. Mol Ther Nucleic Acids. 2021; 26:625–36. 10.1016/j.omtn.2021.08.032 34703648 PMC8517098

[3] Shin VY, Chen J, Cheuk IW, Siu MT, Ho CW, Wang X, Jin H, Kwong A. Long non-coding RNA NEAT1 confers oncogenic role in triple-negative breast cancer through modulating chemoresistance and cancer stemness. Cell Death Dis. 2019; 10:270. 10.1038/s41419-019-1513-5 30894512 PMC6426882

[4] Zhao J, He L, Yin L. lncRNA NEAT1 Binds to MiR-339-5p to Increase HOXA1 and Alleviate Ischemic Brain Damage in Neonatal Mice. Mol Ther Nucleic Acids. 2020; 20:117–27. 10.1016/j.omtn.2020.01.009 32163893 PMC7066222

[5] Ma P, Pan Y, Yang F, Fang Y, Liu W, Zhao C, Yu T, Xie M, Jing X, Wu X, Sun C, Li W, Xu T, Shu Y. KLF5-Modulated lncRNA NEAT1 Contributes to Tumorigenesis by Acting as a Scaffold for BRG1 to Silence GADD45A in Gastric Cancer. Mol Ther Nucleic Acids. 2020; 22:382–95. 10.1016/j.omtn.2020.09.003 33230443 PMC7533296

[6] Kenneweg F, Bang C, Xiao K, Boulanger CM, Loyer X, Mazlan S, Schroen B, Hermans-Beijnsberger S, Foinquinos A, Hirt MN, Eschenhagen T, Funcke S, Stojanovic S, et al. Long Noncoding RNA-Enriched Vesicles Secreted by Hypoxic Cardiomyocytes Drive Cardiac Fibrosis. Mol Ther Nucleic Acids. 2019; 18:363–74. 10.1016/j.omtn.2019.09.003 31634682 PMC6807307

[7] Pisani G, Baron B. NEAT1 and Paraspeckles in Cancer Development and Chemoresistance. Noncoding RNA. 2020; 6:43. 10.3390/ncrna6040043 33143162 PMC7712271

[8] Zhao W, Li W, Jin X, Niu T, Cao Y, Zhou P, Zheng M. Silencing long non-coding RNA NEAT1 enhances the suppression of cell growth, invasion, and apoptosis of bladder cancer cells under cisplatin chemotherapy. Int J Clin Exp Pathol. 2019; 12:549–58. 31933859 PMC6945077

[9] Wu Y, Yang L, Zhao J, Li C, Nie J, Liu F, Zhuo C, Zheng Y, Li B, Wang Z, Xu Y. Nuclear-enriched abundant transcript 1 as a diagnostic and prognostic biomarker in colorectal cancer. Mol Cancer. 2015; 14:191. 10.1186/s12943-015-0455-5 26552600 PMC4640217

[10] Yamazaki T, Souquere S, Chujo T, Kobelke S, Chong YS, Fox AH, Bond CS, Nakagawa S, Pierron G, Hirose T. Functional Domains of NEAT1 Architectural lncRNA Induce Paraspeckle Assembly through Phase Separation. Mol Cell. 2018; 70:1038–53.e7. 10.1016/j.molcel.2018.05.019 29932899

[11] Cui T, Dou Y, Tan P, Ni Z, Liu T, Wang D, Huang Y, Cai K, Zhao X, Xu D, Lin H, Wang D. RNALocate v2.0: an updated resource for RNA subcellular localization with increased coverage and annotation. Nucleic Acids Res. 2022; 50:D333–9. 10.1093/nar/gkab825 34551440 PMC8728251

[12] Hirose T, Yamazaki T, Nakagawa S. Molecular anatomy of the architectural NEAT1 noncoding RNA: The domains, interactors, and biogenesis pathway required to build phase-separated nuclear paraspeckles. Wiley Interdiscip Rev RNA. 2019; 10:e1545. 10.1002/wrna.1545 31044562

[13] Knutsen E, Harris AL, Perander M. Expression and functions of long non-coding RNA NEAT1 and isoforms in breast cancer. Br J Cancer. 2022; 126:551–61. 10.1038/s41416-021-01588-3 34671127 PMC8854383

[14] Xie Z, Wang Q, Hu S. Coordination of PRKCA/PRKCA-AS1 interplay facilitates DNA methyltransferase 1 recruitment on DNA methylation to affect protein kinase C alpha transcription in mitral valve of rheumatic heart disease. Bioengineered. 2021; 12:5904–15. 10.1080/21655979.2021.1971482 34482802 PMC8806685

[15] Liang Y, Peng Y. Gene body methylation facilitates the transcription of CTSG via antisense lncRNA AL136018.1 in dermatomyositic myoideum. Cell Biol Int. 2021; 45:456–62. 10.1002/cbin.11508 33245176

[16] Ye J, Lin Y, Yu Y, Sun D. LncRNA NEAT1/microRNA-129-5p/SOCS2 axis regulates liver fibrosis in alcoholic steatohepatitis. J Transl Med. 2020; 18:445. 10.1186/s12967-020-02577-5 33228663 PMC7686721

[17] Gao Y, Fang P, Li WJ, Zhang J, Wang GP, Jiang DF, Chen FP. LncRNA NEAT1 sponges miR-214 to regulate M2 macrophage polarization by regulation of B7-H3 in multiple myeloma. Mol Immunol. 2020; 117:20–8. 10.1016/j.molimm.2019.10.026 31731055

[18] Wang L, Xia JW, Ke ZP, Zhang BH. Blockade of NEAT1 represses inflammation response and lipid uptake via modulating miR-342-3p in human macrophages THP-1 cells. J Cell Physiol. 2019; 234:5319–26. 10.1002/jcp.27340 30259979

[19] Zhou D, Gu J, Wang Y, Wu H, Cheng W, Wang Q, Zheng G, Wang X. Long non-coding RNA NEAT1 transported by extracellular vesicles contributes to breast cancer development by sponging microRNA-141-3p and regulating KLF12. Cell Biosci. 2021; 11:68. 10.1186/s13578-021-00556-x 33820555 PMC8022671

[20] Zealy RW, Wrenn SP, Davila S, Min KW, Yoon JH. microRNA-binding proteins: specificity and function. Wiley Interdiscip Rev RNA. 2017; 8. 10.1002/wrna.1414 28130820

[21] Gu D, Ahn SH, Eom S, Lee HS, Ham J, Lee DH, Cho YK, Koh Y, Ignatova E, Jang ES, Chi SW. AGO-accessible anticancer siRNAs designed with synergistic miRNA-like activity. Mol Ther Nucleic Acids. 2021; 23:1172–90. 10.1016/j.omtn.2021.01.018 33664996 PMC7900643

[22] Yoon JH, Jo MH, White EJ, De S, Hafner M, Zucconi BE, Abdelmohsen K, Martindale JL, Yang X, Wood WH 3rd, Shin YM, Song JJ, Tuschl T, et al. AUF1 promotes let-7b loading on Argonaute 2. Genes Dev. 2015; 29:1599–604. 10.1101/gad.263749.115 26253535 PMC4536308

[23] Mukherjee K, Ghoshal B, Ghosh S, Chakrabarty Y, Shwetha S, Das S, Bhattacharyya SN. Reversible HuR-microRNA binding controls extracellular export of miR-122 and augments stress response. EMBO Rep. 2016; 17:1184–203. 10.15252/embr.201541930 27402548 PMC4967961

[24] Davis SM, Sousa J, Vangjeli L, Hassler MR, Echeverria D, Knox E, Turanov AA, Alterman JF, Khvorova A. 2'-O-Methyl at 20-mer Guide Strand 3' Termini May Negatively Affect Target Silencing Activity of Fully Chemically Modified siRNA. Mol Ther Nucleic Acids. 2020; 21:266–77. 10.1016/j.omtn.2020.05.010 32610253 PMC7327867

[25] Zhang H, Wei P, Lv W, Han X, Yang J, Qin S. Long noncoding RNA lnc-DILC stabilizes PTEN and suppresses clear cell renal cell carcinoma progression. Cell Biosci. 2019; 9:81. 10.1186/s13578-019-0345-4 31592114 PMC6775667

[26] Sun K, Zhang G. Long noncoding RNA CASC2 suppresses esophageal squamous cell carcinoma progression by increasing SOCS1 expression. Cell Biosci. 2019; 9:90. 10.1186/s13578-019-0353-4 31728180 PMC6842511

[27] Nan S, Wang Y, Xu C, Wang H. Interfering microRNA-410 attenuates atherosclerosis via the HDAC1/KLF5/IKBα/NF-κB axis. Mol Ther Nucleic Acids. 2021; 24:646–57. 10.1016/j.omtn.2021.03.009 33981482 PMC8076652

[28] Chen M, Wei X, Song M, Jiang R, Huang K, Deng Y, Liu Q, Shi D, Li H. Circular RNA circMYBPC1 promotes skeletal muscle differentiation by targeting MyHC. Mol Ther Nucleic Acids. 2021; 24:352–68. 10.1016/j.omtn.2021.03.004 33868781 PMC8027698

[29] Ding L, Liu T, Qu Y, Kang Z, Guo L, Zhang H, Jiang J, Qu F, Ge W, Zhang S. lncRNA MELTF-AS1 facilitates osteosarcoma metastasis by modulating MMP14 expression. Mol Ther Nucleic Acids. 2021; 26:787–97. 10.1016/j.omtn.2021.08.022 34729248 PMC8526484

[30] Ma J, Kong FF, Yang D, Yang H, Wang C, Cong R, Ma XX. lncRNA MIR210HG promotes the progression of endometrial cancer by sponging miR-337-3p/137 via the HMGA2-TGF-β/Wnt pathway. Mol Ther Nucleic Acids. 2021; 24:905–22. 10.1016/j.omtn.2021.04.011 34094710 PMC8141672

[31] Xu L, Liao WL, Lu QJ, Zhang P, Zhu J, Jiang GN. Hypoxic tumor-derived exosomal circular RNA SETDB1 promotes invasive growth and EMT via the miR-7/Sp1 axis in lung adenocarcinoma. Mol Ther Nucleic Acids. 2021; 23:1078–92. 10.1016/j.omtn.2021.01.019 33614250 PMC7875767

[32] Li Z, Shi L, Li X, Wang X, Wang H, Liu Y. RNF144A-AS1, a TGF-β1- and hypoxia-inducible gene that promotes tumor metastasis and proliferation via targeting the miR-30c-2-3p/LOX axis in gastric cancer. Cell Biosci. 2021; 11:177. 10.1186/s13578-021-00689-z 34583752 PMC8480077

[33] Sailer S, Coassin S, Lackner K, Fischer C, McNeill E, Streiter G, Kremser C, Maglione M, Green CM, Moralli D, Moschen AR, Keller MA, Golderer G, et al. When the genome bluffs: a tandem duplication event during generation of a novel Agmo knockout mouse model fools routine genotyping. Cell Biosci. 2021; 11:54. 10.1186/s13578-021-00566-9 33726865 PMC7962373

